# Spatio-temporal mutation profiles of case-matched colorectal carcinomas and their metastases reveal unique *de novo* mutations in metachronous lung metastases by targeted next generation sequencing

**DOI:** 10.1186/s12943-016-0549-8

**Published:** 2016-10-18

**Authors:** Valentina Kovaleva, Anna-Lena Geissler, Lisa Lutz, Ralph Fritsch, Frank Makowiec, Sebastian Wiesemann, Ulrich T. Hopt, Bernward Passlick, Martin Werner, Silke Lassmann

**Affiliations:** 1 Institute for Surgical Pathology, Medical Center-Faculty of Medicine, University of Freiburg, Breisacherstr. 115A, 79106 Freiburg, Germany; 2 German Cancer Consortium (DKTK) and German Cancer Research Center (DKFZ), Heidelberg, Germany; 3 Faculty of Biology, University of Freiburg, Freiburg, Germany; 4 Department of Medicine I, Medical Center-Faculty of Medicine, University of Freiburg, Freiburg, Germany; 5 Comprehensive Cancer Center Freiburg, Medical Center-Faculty of Medicine, University of Freiburg, Freiburg, Germany; 6 Department of General and Visceral Surgery, Medical Center- Faculty of Medicine, University of Freiburg, Freiburg, Germany; 7 Department of Thoracic Surgery, Medical Center-Faculty of Medicine, University of Freiburg, Freiburg, Germany; 8 BIOSS Centre for Biological Signaling Studies, University of Freiburg, Freiburg, Germany

**Keywords:** Colorectal cancer, Next generation sequencing, Metastases, FFPE

## Abstract

**Background:**

Targeted next generation sequencing (tNGS) has become part of molecular pathology diagnostics for determining RAS mutation status in colorectal cancer (CRC) patients as predictive tool for decision on EGFR-targeted therapy. Here, we investigated mutation profiles of case-matched tissue specimens throughout the disease course of CRC, to further specify RAS-status dynamics and to identify *de novo* mutations associated with distant metastases.

**Methods:**

Case-matched formalin-fixed and paraffin-embedded (FFPE) resection specimens (n = 70; primary tumours, synchronous and/or metachronous liver and/or lung metastases) of 14 CRC cases were subjected to microdissection of normal colonic epithelial, primary and metastatic tumour cells, their DNA extraction and an adapted library protocol for limited DNA using the 48 gene TruSeq Amplicon Cancer Panel^TM^, MiSeq sequencing and data analyses (Illumina).

**Results:**

By tNGS primary tumours were RAS wildtype in 5/14 and mutated in 9/14 (8/9 *KRAS* exon 2; 1/9 *NRAS* Exon 3) of cases. RAS mutation status was maintained in case-matched metastases throughout the disease course, albeit with altered allele frequencies. Case-matched analyses further identified a maximum of three sequence variants (mainly in *APC, KRAS*, *NRAS, TP53*) shared by all tumour specimens throughout the disease course per individual case. In addition, further case-matched *de novo* mutations were detected in synchronous and/or metachronous liver and/or lung metastases (e.g. in *APC, ATM, FBXW7, FGFR3, GNAQ, KIT, PIK3CA, PTEN, SMAD4, SMO, STK11, TP53, VHL*). Moreover, several *de novo* mutations were more frequent in synchronous (e.g. *ATM, KIT, PIK3CA, SMAD4*) or metachronous (e.g. *FBXW7, SMO, STK11*) lung metastases. Finally, some *de novo* mutations occurred only in metachronous lung metastases (*CDKN2A, FGFR2, GNAS, JAK3, SRC*).

**Conclusion:**

Together, this study employs an adapted FFPE-based tNGS approach to confirm conservation of RAS mutation status in primary and metastatic tissue specimens of CRC patients. Moreover, it identifies genes preferentially mutated *de novo* in late disease stages of metachronous CRC lung metastases, several of which might be actionable by targeted therapies.

**Electronic supplementary material:**

The online version of this article (doi:10.1186/s12943-016-0549-8) contains supplementary material, which is available to authorized users.

## Background

Predictive molecular pathology mutation testing in selected genes is a routine diagnostic application in several epithelial tumour entities. This includes extended RAS testing (KRAS and NRAS exons 2,3,4) in advanced colorectal cancer (CRC) patients, which functions as well-established predictive biomarker for resistance to EGFR-targeted therapy (e.g. cetuximab or panitumumab) [1–5]. In addition, BRAF mutation testing is recommended for molecular grading of undifferentiated CRCs or as supportive tool for further molecular classification of microsatellite-instable CRCs [6].

Whilst dideoxy-Sanger sequencing, pyrosequencing or quantitative polymerase chain reaction-based assays were routinely used in the past, targeted next generation sequencing (tNGS) has by now evolved as a robust, time- and cost-efficient technique to accommodate the growing demand for mutation profiling in molecular pathology laboratories. Indeed, there are increasing numbers of reports on the general reliability and applicability of tNGS for mutation analyses of formalin-fixed and paraffin-embedded (FFPE) tissue specimens and/or the generation of tumour-entity specific tNGS gene panels, using different platforms and library preparation approaches [7–12]. In addition, several investigators compared primary colon and/or rectal tumours and metastases by NGS approaches in cohorts of 13 cases [13], 15 cases [14], 18 cases [15], 20 cases [16], 24 cases [17], 34 cases [18] or >400 cases [19]. These studies were based on fresh-frozen tissue specimens [15] or assessment of FFPE tissue specimens by amplicon-based semi-conductor NGS technology [13, 16, 17, 19], or evaluation of fresh-frozen tissue specimens with >70 % tumour cell content by whole exome sequencing [14, 18]. Reference to the otherwise mostly small tissue specimens and limited tumour cells of CRC liver or lung metastases in NGS performance and data interpretation is still sparse. Moreover, few of the previous studies focused on the individual clinico-pathological and molecular characteristics of the investigated cases. One study [13] performed tNGS data analysis of 13 matched pairs of the primary tumour plus each one liver metastasis, reporting a 78 % match of mutations. In another study using semi-conductor NGS technology, mutations in for example APC, KRAS, FBXW7, PIK3CA, BRAF, SMAD4 were concordant between primary CRCs and their metastases, whereas 4 of 24 cases also showed *de novo* mutations in SYNE1, CTNNB1, TP53 and PTEN [17]. Similar findings were found in another study, which examined 17 paired primary and mainly synchronous metastatic CRC tissue specimens in a >400 CRC cohort [19]. In two whole exome sequencing based studies [14, 18], mutation profiles of key CRC associated genes were similar in primary and metastatic tumours by 53 % [14] and 57 % [18], with additional *de novo* mutations in metastases of 47 % [14] and 43 % [18]. No correlations of case-matched tumour specimens to clinico-pathological and molecular characteristics were made.

Taken together, there is hence still a lack of studies which address the question of mutation profiles of CRC tumour progression based on case-matched tissue specimen analyses by another NGS platform using limited numbers of microdissected tumour cells, and case-matched data analyses reflecting the individual clinico-pathological and molecular characteristics of CRCs.

In this study, we therefore established a sequencing-by-synthesis targeted NGS approach for analysis of 48 genes in limited numbers of tumour cells microdissected from different types of FFPE tissue specimens. We applied this approach to study mutation profiles of 14 CRC cases with 70 matched tissue specimens from primary tumours and corresponding synchronous and metachronous liver and/or lung metastases. We took into account clinical (treatment regimens, disease course, e.g. synchronous and metachronous metastases), pathological (e.g. primary tumour location, histotype) and molecular (e.g. microsatellite-status, CpG island methylator phenotype) parameters. By this approach, we confirm conservation of predictive RAS mutation status throughout the disease course and identify *de novo* mutations associated with either synchronous or metachronous lung metastases.

## Methods

### Patients and tissue specimens

For establishing the novel tNGS approach, matched Fresh-Frozen (FF) and Formalin-fixed and Paraffin-embedded (FFPE) tissue specimens of 3 non-small cell lung, breast and colorectal carcinomas were selected as technical testing cohort. For further validation, FFPE tissue specimens of two breast cancers (*n* = 4 histologic lesions) and two CRC cases (*n* = 7, including normal epithelium, primary tumour, liver and lung metastases) were selected as technical validation cohort (Additional file 1: Text S1, Table S1).

For analysis of case-matched disease course tissue specimens of CRC patients, FFPE tissue specimens were obtained from 14 CRC patients with synchronous and/or metachronous liver and/or lung metastases (total 70 tissue specimens) undergoing surgery at the Medical Center - University of Freiburg, Germany. In case of multiple lesions at one surgically addressed disease time point (e.g. two lesions in distinct liver segments or two lesions of lung metastases in the right and left lung), these were analyzed individually. Processed tissue specimens hence included normal colonic epithelium dissected from the tumour-free resection margins, primary tumours derived from central tumour areas of resection specimens as well as metastatic liver or lung lesions of resection specimens. All tissue specimens were re-classified and marked by a qualified pathologist (LL) for microdissection and tumour cell content on newly prepared H&E sections serial to those for microdissection.

**Fig. 1:**
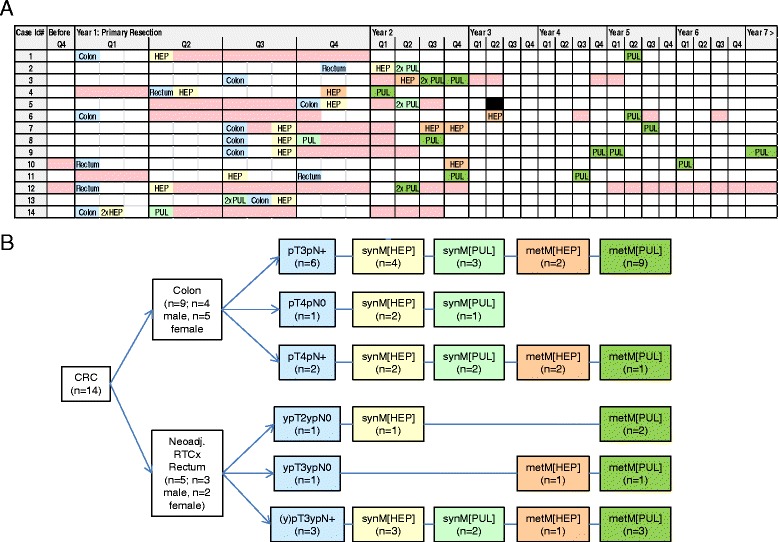
Clinico-pathological data of the colorectal carcinoma patient cohort. **a** The table graph displays the time course and occurrence of resected primary tumours and synchronous or metachronous liver or lung metastases included in the study from 14 patients. **b** The diagram presents the 70 tissue specimens analyzed from the 14 CRC cases according to primary tumour site, classification and metastases. Blue = primary tumours, yellow = synchronous liver metastases, light green = synchronous lung metastases, orange = metachronous liver metastases, dark green = metachronous lung metastases, pink boxes = treatment regimens, black = death of patient. Refer to main text and Table 1 for further clinico-pathological data. Q1-Q4 = “quarterly year”; neoadj. RTCx = neoadjuvant radio-/chemotherapy

Definition of synchronous versus metachronous metastases was according to resection with > 6 months post primary tumour resection. Clinico-pathological parameters - including treatment regimens - of the cases are given in Fig. 1 and Table 1. Note that rectal carcinomas receiving neoadjuvant chemotherapy prior to resection of the primary tumors (4/5 cases) are hence classified with “ypTNM”. Note also that different treatment regimens were given to each case throughout the individual complex disease courses. Therefore, unless otherwise specified, data was analyzed in truly case-matched approach addressing each individual patient.

**Table 1:** Summary of the clinico-pathological data of the colorectal cancer patient cohort

Case ID#	Gender	Age	Location	Date resected	Tissue specimens	Sample ID #	T	Number	Histo-type	G	MSI	CIMP	Treatment
1	m	71	Colon right	03/04	NO	1							Ardalan
				03/04	PT	2	pT3	pN1 (3/20)	NOS	G3	neg	neg	
				05/04	synM[HEP]	3							
				04/08	metM[PUL]	5							
2	f	65	Rectum	12/09	NO	6							Not available
				12/09	PT	7	pT3	pN1 (3/20)	NOS	G3	neg	neg	
				02/10	synM[HEP]	8							
				04/10	synM[PUL]	9							
				06/10	synM[PUL]	11							
3	m	51	Colon left	09/07	NO	13							FOLFOXIRI, FOLFOXIRI/Bevacizumab, Xeloda/Bevacizumab, CAPIRI, Mitomycin, FOLFOX
				09/07	PT	14	pT3	pN1 (1/14)	NOS	G2	neg	neg	
				05/08	metM[HEP]	15							
				09/08	metM[PUL]	16							
				09/08	metM[PUL]	17							
				11/08	metM[PUL]	18							
4	m	64	Rectum^a^	04/05	NO	21							FOLFOX, 5-FU/Mitomycin, FOLFIRI
				04/05	PT	22	ypT3	ypN2 (5/24)	NOS	G2	neg	neg	
				04/05	syn[HEP]	25							
				11/05	metM[HEP]	23							
				02/06	metM[PUL]	49							
5	f	57	Colon left	10/12	NO	28							3x FOLFOXIRI/Bevacizumab
				10/12	PT	50	pT4a	pN2a (6/35)	NOS	G2	neg	neg	
				10/12	synM[HEP]	29							
				04/13	synM[PUL]	32							
				05/13	synM[PUL]	32							
6	f	67	Colon left	03/06	NO	34							3x FOLFIRI/Bevacizumab, FOLFOX
				03/06	PT	36	pT3	pN2 (4/16)	NOS	G2	neg	pos	
				04/08	metM[HEP]	38							
				05/10	metM[PUL]	39							
7	f	35	Colon left	07/07	NO	40							FOLFOX4
				07/07	PT	41	pT4	N1 (2/31)	NOS	G2	neg	neg	
				07/07	synM[HEP]	42							
				07/08	metM[HEP]	44							
				12/08	metM[HEP]	45							
				07/11	metM[PUL]	47							
8	f	50	Colon left	07/10	NO	54							FOLFOXIRI, FOLFOXIRI/Bevacizumab
				07/10	PT	55	pT3	pN1 (1/14)	NOS	G2	neg	neg	
				08/10	synM[HEP]	56							
				10/10	synM[PUL]	58							
				07/11	metM[PUL]	59							
9	f	62	Colon left	07/05	NO	61							Xeloda
				07/05	PT	62	pT3	pN1 (2/12)	NOS	G2	neg	neg	
				09/5	synM[HEP]	63							
				11/08	metM[PUL]	64							
				01/09	metM[PUL]	66							
				02/13	metM[PUL]	68							
10	m	51	Rectum^a^	01/04	NO	70							RCTx
				01/04	PT	71	ypT3	ypN0 (0/15)	NOS	G2	neg	neg	
				10/05	metM[HEP]	73							
				02/09	metM[PUL]	74							
11	f	44	Rectum^a^	12/07	NO	75							FOLFIRI/Bevacizumab
				12/07	PT	76	ypT2	ypN0 (0/4)	NOS	G2	neg	neg	
				07/07	synM[HEP]	77							
				11/08	metM[PUL]	78							
				07/10	metM[PUL]	80							
12	m	48	Rectum^a^	01/11	NO	87							RCTx, Xeloda, FOLFIRI/Bevacizumab, FOLFIRI
				01/11	PT	88	ypT3	ypN1b (2/5)	NOS	G2	neg	neg	
				04/11	synM[HEP]	89							
				04/12	metM[PUL]	91							
				05/12	metM[PUL]	92							
13	m	61	Colon left	09/03	NO	94							Not available
				09/03	PT	95	pT3	pN1 (2/22)	Muc.	G3	neg	pos	
				09/03	synM[HEP]	97							
				07/03	synM[PUL]	98							
				07/03	synM[PUL]	99							
14	m	67	Colon right	02/14	NO	100							2x FOLFOXIRI/Bevacizumab, FOLFIRI/Bevacizumab
				02/14	PT	101	pT4b	pN0 (0/28)	NOS	G2	neg	neg	
				02/14	synM[HEP]	102							
				02/14	synM[HEP]	103							
				04/14	synM[PUL]	104							

*No* normal colonic epithelium, *PT* primary tumour, *synM[HEP]* synchronous liver metastasis, *metM[HEP]* metachronous liver metastasis, *synM[PUL]* synchronous lung metastasis, metM[PUL] metachronous lung metastasis

^a^rectal carcinoma patients received neoadjuvant treatment. The primary anatomic site for colon carcinomas is given with “location”

The use of tissue specimens had been approved by the local ethics Institution (#251/04, #66/07 and #191/13, Ethik-Kommission, University of Freiburg, Germany).

### Microdissection, DNA isolation and DNA quality control

For fresh-frozen tissue specimens, 10 to 20 μm serial sections were cut from each tissue specimen for manual microdissection of normal or tumour cells under morphological control, respective microscope using fine needles. DNA isolation was with the QIAamp Micro Kit, according to the manufacturer’s protocol (Qiagen, Hilden, Germany).

For FFPE tissue specimens, 5 to 10 μm serial sections were cut from each tissue specimen for manual microdissection of normal or tumour cells under morphological control, respective microscope using fine needles. From the testing and validation tissue specimens, DNA isolation was with the QIAamp FFPE DNA Kit, according to the manufacturer`s protocol (Qiagen, Hilden, Germany). For the disease course CRC tissue specimens, DNA was isolated using the Allprep DNA/RNA FFPE Kit according to the manufacturer’s instructions (Qiagen, Hilden, Germany).

For each sample, DNA concentration and purity was determined using the Nanodrop-8000 (ND1000, Peqlab, Erlangen, Germany) and the DNA quality was measured in triplicate using the “FFPE QC Kit” according to the manufacturer`s protocols (Illumina, San Diego, USA). For the latter, the average difference of Cycle-treshold (Ct) values between the DNA samples and a positive DNA control were calculated as relative “QC value”, with “<2” suggested as a valid DNA sample for library preparation by the manufacturer.

### MSI and CIMP analyses of CRC tissue specimens

For MSI testing, a previously published multiplex PCR protocol was used with slight modifications [20]. The CpG island methylator phenotype (CIMP) status was determined by pyrosequencing addressing methylation of five CIMP-related genes (*RUNX3, CACNA1G, EPM2AIP1/MLH1, NEUROG1,CRABP1*) with modifications to previously published protocols [21–23].

### Library preparation and sequencing

Initially, libraries of the technical testing cohort were prepared with the TruSeq Amplicon Cancer Panel (TSACP), covering 225 amplicons of 48 cancer associated genes, following exactly the manufacturer’s protocol (Illumina, San Diego, USA). Since this did not yield reliable libraries for the different types of FFPE tissue specimens (Additional file 1: Table S1A), modifications to the library protocol were made to the first hybridization step with oligo pools and to subsequent PCR cycling parameters (see Additional file 1: Table S1B). The comparison of the performance of these different library protocols is presented for the technical testing and validation cohorts (Additional file 1: Figure S1).

The selection of specific library protocols according to DNA amount and quality for the 70 DNA samples of the CRC study cohort is provided together with the tumour cell content in Additional file 1: Table S2).

Libraries were examined for quantity and quality using the Bioanalyzer 2100 system (Agilent Technologies, Santa Clara, USA) and subjected to clean up, normalization and pooling according to the manufacturer (Illumina, San Diego, USA). MiSeq sequencing runs were performed with each 16–20 pooled libraries using the MiSeq Reagent Kit v2 (300 cycle) (Illumina, San Diego, USA) and paired-end sequencing with 2 × 151 bp.

### Targeted NGS data analysis and validation of detected variants

Established software tools associated with MiSeq technology were used, including Real Time Analysis, MiSeq Reporter analysis and VariantStudio (all Illumina, San Diego, USA). For variant filtering, a 10 % cut-off of allele frequency, with exclusion of synonymous variants and those at a population frequency >5 % (i.e. common sequence variants in KDR p.Q472H, KIT p.M541L and TP53 p.P72R are not reported in text, tables and figures) were used. The Integrative Genomics Viewer (IGV; Broad Institute, UK) [24, 25] was used to visualize read alignments and presence of variants against the reference genome (hg39).

Data sets of normal colonic epithelium were subtracted from those of case-matched tumour (primary, metastases), yielding somatic sequence variants exclusively. *De novo* mutations in metastases were obtained by subtracting the primary tumour data sets from those of case-matched metastases. All detected sequence variants are provided in Additional file 2: Table S4.

Validation of selected somatic variants was done for 27 DNA samples of 8 CRC cases by dideoxy sequencing according to SOP-driven molecular pathology protocols or for TP53 as described previously [26] (Additional file 1: Table S3). All primer sequences are available upon request.

## Results

### Establishment of library preparation from DNA samples derived from different origin tissue specimens

We first tested the standard tNGS library protocol for different types of matched fresh-frozen (FF) and FFPE tissue specimens from a technical testing cohort of 3 pairs of non-small-cell lung (NSCLC), colorectal (CRC) and breast carcinomas (Additional file 1: Figure S1A, Table S1A). To allow tNGS analysis also from low input and poorer DNA quality, we adapted the library protocol (oligo hybridization, PCR cycling conditions) using DNA samples derived from a technical validation cohort of FFPE tissue specimens of two CRC cases (*n* = 7, including normal epithelium, primary tumour, liver or lung metastases) and two breast carcinomas (*n* = 4, different histologic lesions) (Additional file 1: Figure S1B, Table S1B).

Thereby, modification of the library protocol allowed analysis of different types of FFPE tissue specimens, including those with limited DNA amounts from few microdissected tumour cells. Hence, this also enables analysis of limited viable tumour cells from CRC tissue specimens post neoadjuvant therapy or from liver metastases with marked necrosis.

### Targeted NGS of CRC cases with longitudinal disease course tissue specimens

We next applied the new tNGS approach to profile the tissue specimens obtained throughout the individual disease course of 14 CRC patients, each case presenting with specific clinico-pathological parameters, which were included in tNGS data evaluation and interpretation. From each case, normal epithelium, primary tumour and at least two distant metastases were selected for mutation profiling, resulting in *n* = 70 DNA samples in total (Fig. 1, Table 1, Additional file 1: Table S2).

**Fig. 2:**
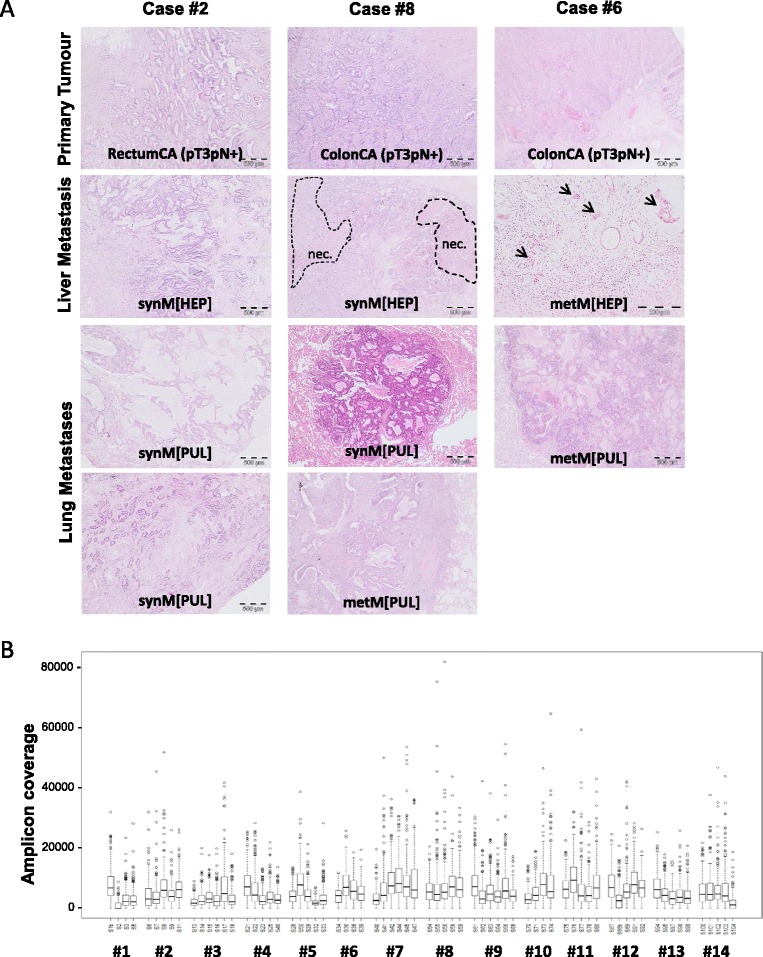
Morphology and tNGS amplicon coverage of investigated primary tumours and distant metastases of the CRC cases. **a** The panels depict H&E sections of case-matched primary tumours, and synchronous and/or metachronous liver or lung metastasis of three representative cases. Note areas of necrosis (“nec”; e.g. case #8, synM(HEP)) and widely distributed clusters of few tumor cells (e.g. case #6, metM(HEP)), necessary for interpretation of gene mutations with lower allele frequencies. All panels same magnification 2.5x except case #6 metM(HEP) being 10x. **b** Summary of the amplicon coverage for all 70 investigated DNA samples of the CRC cohort. Refer to the main text, Table 1 and Additional file 1: Table S2 for further details

As expected from the size and histology of the lesions of primary tumour and metastatic sites (Fig. 2a), the DNA samples derived from the microdissected tumour cells showed a wide range of DNA concentrations (5 to 452 ng/μl, reflecting the maximum DNA of enriched tumour cells from the partially small tissue specimens of metastatic lesions; mean tumour cell content of enriched samples = 69 %, with 4 DNA samples from cases #1, #6, #13 showing only 10–30 % tumour cell enrichment; Additional file 1: Table S2) and quality scores (QC range 0.1 to 3, with a QC value >2 in 24/70 DNA samples), therefore requiring adjusted tNGS library preparation according to the newly established protocols (Additional file 1: Table S2). Thereby, all 70 DNA samples yielded sufficient libraries, resulting in mean amplicon coverage of 5767x (range 1003x to 9517x) upon sequencing (Fig. 2b).

With a cut-off of 10 % allele frequency for variant calling, the most frequently mutated genes, respective sequence variants occurring in at least 2 tissue specimens of primary tumour and/or liver and/or lung metastases were: TP53 (10/14 cases), APC (8/14 cases), KRAS (8/14 cases), SMAD4 (2/14 cases) as well as FGFR3 (1/14 cases) and PIK3CA (1/14 cases) and NRAS (1/14 cases) (Table 2). See also Additional file 2: Table S4 for all detected sequence vairants.

**Table 2:** Summary of genes mutated in 2 or more case-matched tumour sites throughout disease course

Case ID#	Gene	PT	syn M[HEP] 1	syn M[HEP] 2	syn M[PUL] 1	syn M[PUL] 2	met M[HEP] 1	met M[HEP] 2	met M[PUL] 1	met M[PUL] 2	met M[PUL] 3
1	KRAS	G12D	G12D						G12D		
	TP53	R342*	WT						R342*		
2	APC	Q1338*	Q1338*		Q1338*	Q1338*					
	KRAS	G12V	G12V		G12V	G12V					
	SMAD4	R361C	R361C		R361C	R361C					
3	APC	WT					R1450*		R1450*	WT	R1450*
		R876*					R876*		R876*	R876*	R876*
	KRAS	G12V					G12V		WT	G12V	G12V
	PIK3CA	WT					K111E		WT	WT	K111E
	TP53	R196*					R196*		R196*	R196*	R196*
4	TP53	R337C	R337C				R337C		R337C		
		G199V	G199V				G199V		G199V		
5	APC	P1319Lfs*	P1319Lfs*		P1319Lfs*	P1319Lfs*					
	KRAS	G12A	G12A (9.7 % AF)		G12A	G12A					
	PIK3CA	E542K	E542K		E542K	E542K					
	TP53	G244C	G244C		G244C	G244C					
6	APC	Q1367*					WT		Q1367*		
	KRAS	G12V					WT		G12V		
	SMAD4	Q250*					WT		Q250*		
7	TP53	V157F	V157F				V157F	V157F	V157F		
8	KRAS	G13D	G13D		G13D				G13D		
	TP53	R248Q	R248Q		R248Q				R248Q		
9	APC	R1114*	R1114*						R1114*	R1114*	R1114*
	TP53	R282W	R282W						R282W	R282W	R282W
10	APC	T1556Nfs*					T1556Nfs*		T1556Nfs*		
	FGFR3	P718S					P718H		P718S		
	TP53	V157G					V157G		V157G		
11	TP53	R273C	R273C						R273C	R273C	
12	APC	WT	L1488Tfs*						L1488Tfs*	L1488Tfs*	
	KRAS	G12A	G12A						G12A	G12A	
13	ABL1	WT	WT		P315S	P315S					
	APC	R876*	WT		R876*	WT					
	APC	E1284*	WT		E1284*	E1284*					
	ERBB4	WT	WT		D300N	D300N					
	KRAS	G12D	p.G12V		G12V (5.6 % AF)	G12V					
14	APC	T1430Pfs*	T1430Pfs*	T1430Pfs*	T1430Pfs*						
	NRAS	Q61R	Q61R	Q61R	Q61R						
	TP53	R175H	R175H	R175H	R175H						

*AF* allele frequency

### RAS mutation status is maintained in case-matched primary CRCs and their metastatic sites

We next examined the RAS status throughout the disease course of the individual CRC cases.

Of the 14 cases, 8/14 (57 %) exhibited *KRAS* (exon 2, codon 12 or 13) mutations in the primary tumour and 1/14 (7 %) a *NRAS* (exon 3, codon 61) mutation (Table 3). These RAS mutations were detectable throughout the disease course in each resected tumour lesion in 7/9 (78 %) mutated cases. Thereby, the allele frequency of the RAS mutations varied between primary tumour, liver and/or lung metastases. In two cases, the allele frequencies were below 10 % in the synchronous liver metastasis (9.7 %, case #5) and in one of the two synchronous lung metastastic lesions (5.6 %, case #13). In cases #3 and #6, the *KRAS* p.G12V mutation was not detected in the metachronous lung and liver metastasis even at a cut-off level of >1 % allele frequency. The drop of allele frequency in mainly small metastatic lesions most likely relates to the limited or only single distributed (e.g. case #6 liver metastasis, Fig. 2a) tumour cells available for microdissection.

**Table 3:** The RAS mutation status is maintained throughout disease course

Case ID#	RAS Mut.	PT	syn M[HEP] 1	syn M[HEP] 2	syn M[PUL] 1	syn M[PUL] 2	met M[HEP] 1	met M[PUL] 1	met M[PUL] 2	met M[PUL] 3
1	KRAS G12D	55.59	97.28					16.77		
2	KRAS G12V	53.59	89.98		47.32	32.82				
3	KRAS G12V	21.92					45.26	0 and 21.22	34	
4	WT	0	0				0	0		
5	KRAS G12A	24.18	9.7^a^		17.72	36.04				
6	KRAS G12V	22.85					0	26.73		
7	WT	0	0				0, 0			
8	KRAS G13D	34.16	50.44		31.43			12.85		
9	WT	0	0					0	0	
10	WT	0					0	0		
11	WT	0	0					0		
12	KRAS G12A	99.74	56.72					34.15	19.63	
13	KRAS G12D, G12V	19.83 (G12D)	16.44 (G12V)		5.62^a^ and 25.72 (G12V)					
14	NRAS Q61R	44.31	66.91 and 44.74		46.17					

The table summarizes the RAS status and mutated allele frequency (%) throughout the disease course of 9/14 mutated CRC cases. If two metastatic lesions were resected at the same time, the RAS status is given for both

^a^= sequence variant detected at below 10 % allele frequency

Finally, none of the 5 cases with wildtype RAS in the primary tumour subsequently developed RAS mutations in the EGFR-targeted therapy relevant “hotspot” codons (Table 3).

### Mutation profiles of CRC patients throughout the disease course

To determine mutation profiles of tumour sites, respective locations of disease progression, we first examined the NGS data sets of all 70 DNA samples in a case-mixed manner.

This revealed tumour site associated shared sequence variants in 1) *APC, PDGFRA, PTEN* for primary colon and/or rectal tumours, 2) *ATM, TP53* for synchronous liver metastases, 3) *FGFR3* and *TP53* for metachronous liver metastases, 4) *ABL1, APC, ATM, ERBB4* and *SMAD4* for synchronous lung metastases, and 5) *APC* and *TP53* for metachronous lung metastases (Table 4).

**Table 4:** Case-mixed analysis of tumour site specific mutations

Gene	Variant	PT - Colon	PT - Rectum	syn M[HEP]	syn M[PUL]	met M[HEP]	met M[PUL]
ABL1	p.315S	0	0	0	2	0	0
APC	p.R876*	2	0	0	0	0	3
	p.R1114*	0	0	0	0	0	4
	p.E1284*	0	0	0	2	0	0
	p.Q1338*	0	0	0	2	0	0
ATM	p.A1812V	0	0	2	0	0	0
ERBB4	p.D300N	0	0	0	2	0	0
FGFR3	p.T722I	0	0	0	0	2	0
PDGFRA	p.P577S	1	1	0	0	0	0
PTEN	p.Q171*	2	0	0	0	0	0
	p.H185Y	1	1	0	0	0	0
SMAD4	p.R361C	0	0	0	2	0	0
TP53	p.W91*	0	0	0	0	0	2
	p.175H	0	0	2	0	0	0
	p.V157F	0	0	0	0	2	0
	p.R196*	0	0	0	0	0	3
	p.R282W	0	0	0	0	0	3
	p.R273C	0	0	0	0	0	2

The table presents the gene variants detected in at least 2 samples per tumour site further to KRAS and NRAS, as analyzed in a case-mixed manner and with exclusion of known SNPs

Hence, this case-mixed analysis suggests some gene sequence variants specific for liver or lung metastases, but gives no information on whether or not these were already present in e.g. the primary tumour of individual CRC patients.

### Case-matched mutation profiles throughout disease course reveal distinct *de novo* mutations in synchronous versus metachronous lung metastases

Since the clinico-pathological parameters and disease course are individual for each CRC patient (e.g. neoadjuvant treatment in 4/5 rectal tumours as well as adjuvant treatment regimens), we next analyzed the case-matched mutation profiles, specifically focusing on the late(st) stages of lung metastasis.

**Fig. 3:**
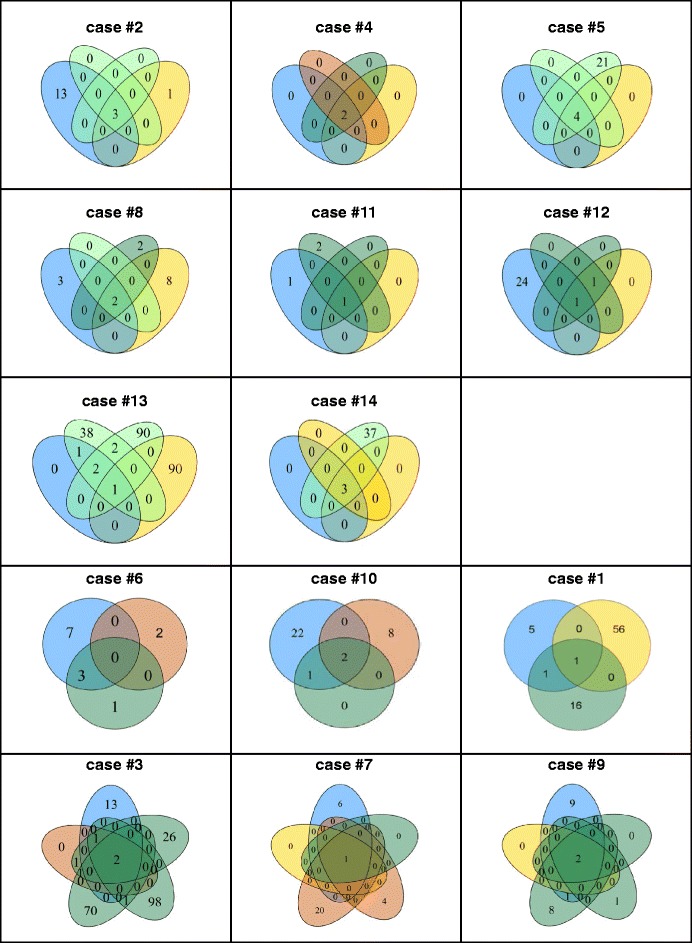
Case-matched shared mutations in primary tumours and distant metastases of CRC cases. The panels depict Venn diagrams of variants detected per case in primary tumour (*blue*), synchronous (*yellow*) and metachronous (*orange*) liver metastasis as well as synchronous (light green) and metachronous (*dark green*) lung metastases. Numbers represent the number of sequence variants being shared (overlaps) or separate for a specific CRC tissue specimen. Note that CRC cases #4, #5 and #14 exhibited mutations in the primary tumour, which were then also all detected in distant metastases and are hence only depicted in the overlaps. Note three patterns of CRC cases: type I = cases #2, 4, 5, 8, 11, 12, 13, 14; type II = # 6, 10, 1 and type III = #3, 7, 9. These three types did not correlate with the specific clinico-pathological or molecular features. Refer to main text for details

As shown by the Venn diagrams for each CRC case in Fig. 3, generally few distinct mutations (maximum of 3, including APC, KRAS, NRAS, PIK3CA, SMAD4 and/or TP53) were shared between all case-matched DNA samples (primary tumour, synchronous and/or metachronous liver and/or lung metastases) of an individual CRC case (Table 2). Taking into account the individual clinico-pathological parameters, there was no association of mutation profiles to primary tumour site (left colon, right colon, rectum) or histology, tumour stage, grading or molecular subtype (Fig. 3).

**Fig. 4:**
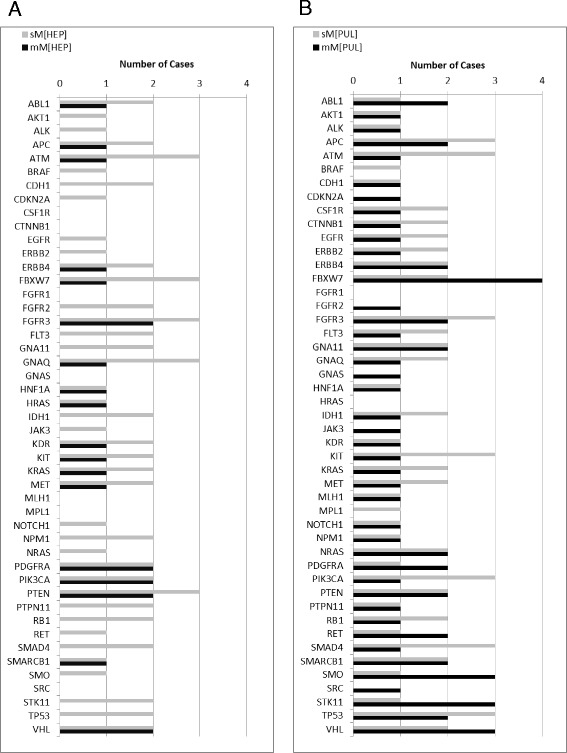
*De novo* mutations detected in synchronous and metachronous liver and lung metastases. **a** The graph presents the number of CRC cases with *de novo* mutations detected for all investigated 48 genes in synchronous (light grey; sM[HEP]) and metachronous (dark grey; mM[HEP]) liver metastases. **b** The graph presents the number of CRC cases with *de novo* mutations detected for all investigated 48 genes in synchronous (light grey; sM[PUL]) and metachronous (dark grey; mM[PUL]) lung metastases

However, interestingly numerous case-matched *de novo* mutations occurred in liver (cases #1, 2, 6, 7, 8, 10, 13) and lung (cases #1, 2, 3, 6, 7, 8, 9, 10, 11, 12) metastases, which were not present in the case-matched primary tumours (Fig. 3): Case-matched analysis and comparison of synchronous (*n* = 12 DNA samples of 11 CRC cases) versus metachronous (*n* = 6 DNA samples of 5 CRC cases) liver metastases yielded 4/12 (33 %) synchronous versus 4/6 (67 %) metachronous liver metastases with unique *de novo* mutations. Thereby, the *de novo* mutation profiles ranged from 1–90 and 2–20 variants in synchronous as compared to metachronous liver metastases, including mutations in *FBXW7, FGFR3, GNAQ* and *PTEN* occurring in more than 2 cases with synchronous liver metastases (Figs. 3 and 4a).

In contrast, case-matched analysis and comparison of synchronous (*n* = 8 of 5 CRC cases) and metachronous (*n* = 16 of 10 CRC cases) lung metastases revealed a broad mutation profile in both synchronous (range of variants: 21–90) and metachronous (range of variants: 1–98) lung metastases (Figs. 3 and 4b). Thereby, synchronous lung metastasis more frequently showed mutations in *ATM, KIT, PIK3CA* and *SMAD4* (each with 3/5 cases as compared to 1/5 cases with metachronous liver metastases). Moreover, metachronous lung metastases showed exclusive mutations in *CDKN2A, FGFR2, GNAS, JAK3* and *SRC* (each 1/10 cases as compared to 0/5 cases with synchronous lung metastases) as well as frequent mutations in *FBXW7* (4/10 cases; synchronous: 2/5 cases), SMO (3/10 cases; synchronous: 1/5 cases) and *STK11* (3/10 cases; synchronous: 1/5 cases).

As above, also the identified *de novo* mutations in lung metastases were not associated with clinico-pathological parameters. Thereby, neither the number of *de novo* mutations and/or the genes affected by *de novo* mutations were directly linked to specific prior treatment regimens, as summarized in Table 5.

**Table 5:** *De novo* gene mutations in synchronous and metachronous lung metastasis in relation to treatment regimens

Case ID #	syn/met	Year of occurrence post PT	Therapy regimen	*De novo* mutations
1	met	5	Ardalan	ABL1, FGFR3, KRAS, NRAS, RET, SMARCB1, STK11, TP53, VHL
3	met	2	FOLFOXIRI, FOLFOXIRI/Bevacizumab, Xeloda/Bevacizumab, CAPIRI, Mitomycin, FOLFOX	AKT1, ALK, APC, ATM, CSF1R, EGFR, ERBB2, ERBB4, FBXW7, FGFR2, FGFR3, FLT3, GNA11, GNAQ, HNF1A, IDH1, JAK3, KDR, KIT, KRAS, MET, MLH1, NOTCH1, NPM1, NRAS, PDGFRA, PIK3CA, PTEN, PTPN11, RB1, RET, SMAD4, SMO, SRC, STK11, TP53, VHL
4	met	2	FOLFOX, 5-FU/Mitomycin, FOLFIRI	No *de novo* mutations
5	syn	2	3x FOLFOXIRI/Bevacizumab	APC, ATM, FBXW7, FGFR3, KIT, KRAS, PIK3CA, PTEN, RB1, SMAD4, TP53, VHL
6	met	5	3x FOLFIRI/Bevacizumab, FOLFOX	SMO
7	met	5	FOLFOX4	No *de novo* mutations
8	syn,met	1,2	FOLFOXIRI, FOLFOXIRI/Bevacizumab	FBXW7
9	met	4,5,7	Xeloda	STK11, VHL, SMO, FBXW7, ERBB4, GNA11, PDGFRA
10	met	6	RCTx	No *de novo* mutations
11	met	2,4	FOLFIRI/Bevacizumab	PTEN, FBXW7
12	met	2	RCTx, Xeloda, FOLFIRI/Bevacizumab, FOLFIRI	APC
14	syn	1	2x FOLFOXIRI/Bevacizumab, FOLFIRI/Bevacizumab	ABL1, AKT1, APC, ATM, BRAF, CSF1R, CTNNB1, EGFR, ERBB2, ERBB4, FGFR3, FLT3, GNA11, GNAQ, IDH1, KIT, MET, PIK3CA, SMAD4, SMARCB1, TP53, VHL

The table summarizes *de novo* mutations per case in relation to occurrence of metastasis and treatment regimens. *RCTx* radio/chemotherapy, *Syn* synchronous, *met* metachronous. Note that there is no direct correlation between specific treatment regimens and the number of *de novo* mutations and/or genes affected by *de novo* mutations. Refer to main text for details

Hence, these data allow case-matched and tumour site location specific identification of gene mutations involved in disease progression of individual CRC cases.

### Multiple lesions of lung, but not liver metastases resected at one time point may differ in the mutation profile

**Fig. 5:**
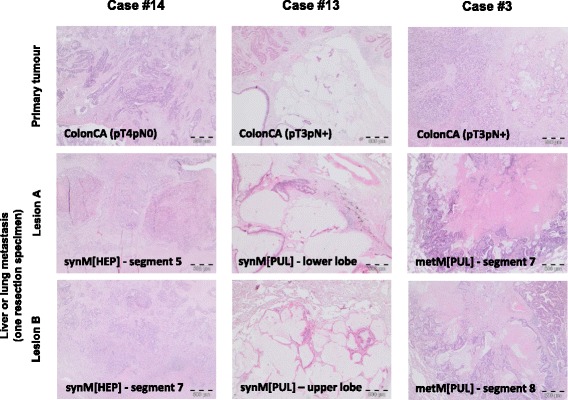
Morphology of case-matched separate CRC metastatic liver or lung lesions resected at one time point with similar or distinct mutation profiles. The panels show H&E stainings of primary tumours and their associated resected liver or lung metastases, from which each two topographically distant located separate lesions (labelled lesion A and lesion B) were individually analyzed by tNGS. Case # 14 showed the same mutation profile in the primary tumour and its associated two metastatic liver lesions (within segments 5 and 7). Cases #13 (upper, lower lobes of lung) and cases #3 (segments 7 and 8) each showed only few shared mutations and numerous additional mutations associated with either of the individual lung metastatic lesions, despite similar morphology. Refer to main text for details

Finally, *de novo* mutations in each two separate topographic lesions of liver or lung metastases of one resection in 3 CRC cases (#3, #13, #14) were examined (Fig. 5):

Exactly the same mutation profile (mutations in *APC, NRAS, TP53*) were detected in two lesions (liver segments 5 and 7) of the liver metastasis of case #14.

In contrast, in two lesions (upper, lower lobes) of the synchronous lung metastasis of case #13 shared mutations occurred in *ABL1, APC, ATM* and *ERBB4*, but this was accompanied by 39 (“lower lobe left”) and 90 (“upper lobe left”) additional variants for each separate lesion. Similarly, in two lesions (lung segments 7 and 8) of the metachronous lung metastasis of case #3 shared mutations were detected in APC (*n* = 2), FGFR2 (*n* = 1) and TP53 (*n* = 1), which was associated with 70 (“segment 7”) and 98 (“segment 8”) additional variants. Thereby, the different mutation profiles of each the two lung metastatic lesions per resection were seen for the mucinous (case #13) and the tubular/NOS (case #3) CRC case (Fig. 5).

## Discussion

This study comprehensively evaluated the feasibility and outcome of targeted next generation sequencing of case-matched colorectal cancer (CRC) Formalin-fixed and Paraffin-embedded tissue specimens in a close morphological context.

The study describes a novel protocol which allows targeted next generation sequencing (tNGS) even from low input and/or poorer quality DNA derived from microdissected tumour cells of archival tissue specimens using a sequencing-by-synthesis tNGS approach.

Moreover, the present study shows that mutation profiling of CRC, respective investigation of “driver mutations”, intratumoural heterogeneity and tumour evolution need to be performed in a fully case-matched (i.e. taking into account individual treatment regimens, including e.g. neoadjuvant therapy of rectal cancers prior to resection of the primary tumour) and morphology-controlled manner: The spectrum of apparent “driver mutations” of e.g. lung metastasis were different upon evaluation of all lung metastases in a case-mixed manner as compared to analysis of true “*de novo*” mutations of lung metastases in a case-matched manner. In addition, the present study reveals an obvious difference of the mutation profile in (case-matched) synchronous and metachronous liver or lung metastases, suggesting tumour heterogeneity and distinct tumour evolution. Hence, the investigative approach of the CRC cohort here focused on those mutations possibly accounting for tumour progression of individual patients more than 2 years post resection of the primary tumour.

The CRC cohort here only included 14 CRC patients, but a total of 70 tissue specimens obtained during case-matched follow-up, respective disease progression, especially resected lung metastases, were investigated in a case-matched manner also for tNGS data analysis and interpretation. Although this appears a very small number of cases, this is in fact similar in other CRC screening studies. For example, a large study of initially 2000 CRC patients, then selected 468 cases for NGS and of these only 19 cases had matched metastases, with 12/19 cases presenting distant metastases [19]. In other studies, 13 cases with mainly liver metastases [13]; 24 cases with either one (*n* = 7), two (*n* = 11) or three (*n* = 6) local or distant metastatic lesions (lymph node, liver, lung, brain) [17]; 16 rectal carcinomas with lymph node (*n* = 13/16), liver (*n* = 1/16) or lung (*n* = 2/16) [16] were included. All of these studies were performed by semi-conductor NGS technology as compared to the present study using a sequencing-by-synthesis tNGS approach. Few studies commented on the morphological aspects of the (microdissected?) tumour specimens in case when allele frequencies drop, which may merely be due to few (enriched?) tumour cells from CRC tissue specimens (e.g. especially in neoadjuvant treated primary rectal tumours or highly necrotic liver metastases). Clearly, statistical sub-group analyses in such a small cohort are not feasible to address all of these clinico-pathological factors. Hence, the present study reports on and presents case-matched tNGS results with associated clinico-pathological data and morphological observations.

In fact, our present study addresses the morphological aspect from different angles: First, by precise microdissection under a microscope using fine needles we clearly eliminate e.g. lymphocyte-rich or necrotic areas from DNA sample preparation. Second, we provide the tumour cell content in terms of the percent of enriched (tumour) cell nuclei, not in terms of tissue area. Third, we report a feasible approach that is applicable to routine molecular pathology diagnostics – the initial diagnostic biopsy or resection specimen is used (not a separate tissue specimen obtained just for molecular analysis by an invasive approach, which is frequently impossible in advanced disease stages of CRC and other solid tumours) and all data interpretation is made with respect to the morphology, as specifically shown for the selected multiple distant metastatic tumour lesions of single resection specimens.

In this setting, we first report on the findings of the RAS mutation status by tNGS in the CRC cohort, with 9/14 cases showing known *KRAS* or *NRAS* mutations. Thereby, mutation status was maintained throughout the disease course albeit with altered allele frequencies. In fact, 2/9 cases showed a RAS mutation in the primary tumour, which was apparently “lost” at first sight in their metastases. Careful evaluation of the morphology clearly showed only few tumour cells available for DNA extraction in serial sections of the tissue specimens, which without morphological controlled enrichment might have been diluted in “non-tumour cell (and necrotic) background”. However, a morphology-adapted tNGS cut-off to lower allele frequencies then indeed showed RAS mutations at <5 % in the 2/5 cases. Similarly, observations were reported in a previous study for *KRAS* and other gene mutations [17], however no reference was made as to the morphology of these lesions, which in liver and lung tend to be highly necrotic with a paucity of tumour cells for enrichment by microdissection.

Hence, the present data validates mutations in genes (e.g. *APC, KRAS, NRAS, TP53*) known to be involved in (sporadic) CRC development and throughout progression [13, 16, 17, 27] with close reference to the clinico-pathological features. In fact, these key mutations, here referring to the exact sequence variant, were not directly associated with the primary tumour location (left or right colon, rectum) and histology, tumour stage, grading or molecular sub-type (Microsatellite-(in)stability; CpG-Island methylator phenotype) in this small cohort. Moreover, in the present study only a maximum of 3 sequence variants were shared between all CRC tumour sites throughout disease progression upon case-matched analyses, whereas broader and new mutation profiles were seen especially upon tumour progression to (synchronous or metachronous) lung metastases. The limited overlap of shared sequence variants between primary CRC and multiple metastatic lesions was not reported before. However, also others showed the same few genes to be similarly affected in primary tumours and associated distant metastases [13, 15, 17, 19].

In addition to the above key genes affected, *de novo* mutations occurring in liver or lung metastases of case-matched tumours in at least 3 cases were in *APC, ATM, FBXW7, FGFR3, GNAQ, KIT, PIK3CA, PTEN, SMAD4, SMO, STK11, TP53* and *VHL*. Thereby, synchronous vs metachronous as well as liver vs lung metastases displayed distinct gene patterns suggesting distinct tumour evolution. Of these, several mutations may be actionable through targeted therapies interfering with the affected signaling pathways, e.g. FBXW7 (Additional file 1: Figure S2) (this study and [14, 16, 17, 28–30]) and its functional consequences on mTOR and PTEN signaling [31–33] and mTOR inhibitors [34]. These data suggest that when looking at advanced disease stages of CRCs, each patient needs to be individually addressed due to the case-specific prior surgical and clinical treatment regimens and associated case-specific molecular progression. Hence, sequential biopsies – if clinically possible – or accompanying liquid biopsies may eventually evolve as supplementary tool to SOP-driven molecular tissue based analyses for defining individual treatment options.

## Conclusion

Together, our present study demonstrates a novel approach for targeted next generation sequencing of routine diagnostic FFPE tissue specimens and thereby again highlights the importance of morphological-controlled tissue specimens. By this, the study identifies only few shared mutations, respective exact sequence variants throughout the disease course of individual colorectal cancer patients, including *KRAS* and *NRAS*. Furthermore, the study shows case-matched *de novo* altered mutation profiles in synchronous vs metachrounous liver and/or lung metastasis. Some of the affected genes - e.g. *FBXW7* and *PTEN* may thereby be further explored for targeted therapy, including validation of findings in larger prospective case series.

## Additional files

Additional file 1:
**Text S1.** Establishment of library preparation from DNA samples derived from different origin tissue specimens. Adaptations of the tNGS library protocol. **Table S1.** Modifications to tNGS library protocol of low input or poorer quality DNA improves sequencing performance. **Table S2.** Details of tissue specimens and associated DNA and library processing data. **Table S3.** Validation of variants detected by tNGS by dideoxy sequencing. **Figure S1.** Quantity and quality of tNGS libraries of the standard and adapted library protocols. **Figure S2.** De novo FBXW7 sequence variants detected in colorectal carcinoma liver and/or lung metastases. (DOCX 556 kb)
Additional file 2: Table S4. Summary file of all detected sequence variants. (XLSX 69 kb)
