# Supplementary material for: Spatio-temporal mutation profiles of case-matched colorectal carcinomas and their metastases reveal unique de novo mutations in metachronous lung metastases by targeted next generation sequencing
Source: Mol Cancer. 2016 Oct 18;15:63. doi: 10.1186/s12943-016-0549-8 (PMC5069823; doi:10.1186/s12943-016-0549-8)

**Additional file 1**

**Supplementary to**

**Spatio-temporal mutation profiles of case-matched colorectal carcinomas and their metastases reveals *de novo* mutations in metachronous lung metastases by targeted Next Generation Sequencing**

Valentina Kovaleva^1,2*^, Anna-Lena Geissler^1,2,3*^, Lisa Lutz^1^, Ralph Fritsch^4,5^, Frank Makowiec^5,6^, Sebastian Wiesemann^5,7^, Ulrich T. Hopt^5,6^, Bernward Passlick ^5,7^, Martin Werner^1,2,5^, Silke Lassmann^1,2,5,8,§^

^1^ Institute for Surgical Pathology, ^4^ Dept. of Medicine I, ^5^ Comprehensive Cancer Center Freiburg, ^6^ Dept. of General and Visceral Surgery, ^7^Dept. of Thoracic Surgery, all Medical Center - University of Freiburg and Faculty of Medicine, University of Freiburg; ^3^ Faculty of Biology, University of Freiburg; ^2^ German Cancer Consortium (DKTK) and German Cancer Research Center (DKFZ), Heidelberg; ^8^ BIOSS Centre for Biological Signaling Studies, University of Freiburg; all Germany; * equal contribution

§**Corresponding author:** Prof. Dr. Silke Lassmann, Institute for Surgical Pathology

Medical Center – University of Freiburg, Breisacherstr. 115A

79106 Freiburg, Tel.: (+49) 0761-270-80620

Email: silke.lassmann@uniklinik-freiburg.de

**Contents:**

1. Text S1. Establishment of library preparation from DNA samples derived from different origin tissue specimens
2. Adaptations of the tNGS library protocol
3. Table S1. Modifications to tNGS library protocol of low input or poorer quality DNA improves sequencing performance.
4. Table S2. Details of tissue specimens and associated DNA and library processing data.
5. Table S3. Validation of variants detected by tNGS by dideoxy sequencing.
6. Table S4. Summary file of all detected sequence variants (separate file)
7. Figure S1. Quantity and quality of tNGS libraries of the standard and adapted library protocols.
8. Figure S2. De novo FBXW7 sequence variants detected in colorectal carcinoma liver and/or lung metastases.

**1. Establishment of library preparation from DNA samples derived from different origin tissue specimens**

We first tested the standard tNGS library protocol for different types of matched fresh-frozen (FF) and FFPE tissue specimens from a technical testing cohort of 3 pairs of non-small-cell lung (NSCLC), colorectal (CRC) and breast carcinomas (Supplementary Figure S1A). Whilst the concentrations of DNA samples from matching FF and FFPE tissue specimens varied as expected according to the available tumour cell content, there were marked differences in the DNA quality, being poor (QC value >2) in the FFPE tissue specimen of the breast carcinoma (Figure 2A). Accordingly, standard library preparation and MiSeq sequencing yielded valid data (library concentration, cluster density, mean coverage) only for NSCLC and CRC, but not for breast carcinoma tissue specimens (Supplementary Figure S1A, Supplementary Table S1).

To allow tNGS analysis also from low input and poorer DNA quality, we adapted the library protocol using DNA samples derived from a technical validation cohort of FFPE tissue specimens of two CRC cases (n=7, including normal epithelium, primary tumour, liver or lung metastases) and two breast carcinomas (n=4, different histologic lesions) (Supplementary Figure S1B, Supplementary Table S1B). First, the standard library protocol was modified for oligo hybridization time from 1 (Standard) to 10 minutes with similar PCR cycling conditions (“Protocol 1”), which did not significantly improve the library yield and subsequent mean amplicon coverage: 9/11 DNA samples had a mean amplicon coverage range from 65x to 1190x. Subsequently, the library protocol was modified for both oligo hybridization (10 min or 20min) and PCR cycling parameters (“Protocol 2” and “Protocol 3”). “Protocol 2” yielded sufficient libraries in 6/7 tested DNA samples and “Protocol 3” in tested 11/11 DNA samples. Accordingly, the two sets of libraries of “Protocol 2” and “Protocol 3” resulted in mean amplicon coverages of >1000x (“Protocol 2” range: 1153x to 1654x; “Protocol 3”: 1095x to 4896x; Supplementary Figure S1B, Supplementary Table S1B).

Thus, modification of the library protocol allows analysis of different types of FFPE tissue specimens, also those with limited DNA amounts from enrichment of few microdissected tumour cells, i.e. also enabling analysis of limited viable tumour cells from CRC tissue specimens post neoadjuvant therapy or from liver metastases with marked necrosis.

**2. Adaptations of the tNGS library protocol.** Modifications to the tNGS library protocol were:

- PROTOCOL 1:

Hybridization: 10min Hybridization

PCR: 95°C/5min; 27 cycles of [95°C/3min, 62°C/30sec, 72°C/1min]; 72°C/5min

- PROTOCOL 2:

Hybridization: 10min Hybridization

PCR: 95°C/5min; 40 cycles of [95°C/1min, 62°C/1min, 72°C/1.5min]; 72°C/7min

- PROTOCOL 3:

Hybridization: 10min Hybridization

PCR: 95°C/5min; 32 cycles of [95°C/45sec, 62°C/45sec, 72°C/1min]; 72°C/5min

- PROTOCOL 4:

Hybridization: 20 min Hybridization

PCR: 95°C/5min; 35 cycles of [95°C/1min, 62°C/1min, 72°C/1min]; 72°C/5min

- PROTOCOL 5:

Hybridization: 20 min Hybridization

PCR: 95°C/5min;40 cycles of [95°C/1min, 62°C/1min, 72°C/1min]; 72°C/5min

**3. Table S1**. **Modifications to tNGS library protocol of low input or poorer quality DNA improves sequencing performance.** The table summarizes the technical performance of different types of tissue specimens upon use of the standard (A) and adapted (B) tNGS library protocols.

| **ID#** | **Tissue Type** | **DNA [ng/µl]** | **QC** | **Input (ng)** | **Libr. Protocol** | **Library [nM]** | **Clusters Pass Filter** | **Mean Coverage** |
| --- | --- | --- | --- | --- | --- | --- | --- | --- |
| **A) Technical testing cohort** | | | | | | | | |
| **NSCLC** | FF | 108 | 0.29 | 150 | Standard | 73.4 | 1,113,878 | 5,321x |
|  | FFPE | 100 | 0.92 | 150 | Standard | 15.1 | 452,106 | 2,110x |
| **Colorectal Cancer** | FF | 198 | 2.1 | 150 | Standard | 22.2 | 480,877 | 2,255x |
|  | FFPE | 263 | 0.88 | 150 | Standard | 30.1 | 570,741 | 2,652x |
| **Breast Cancer** | FF | 65 | 0.67 | 250 | Standard | 1.1 | 77,621 | 358x |
|  | FFPE | 146 | 4.53 | 250 | Standard | 1.0 | 99,667 | 462x |
| **B) Technical validation cohort** | | | | | | | | |
| **Case #5**  **Normal Colon (28)** | FFPE | 50 | 2.2 | 250 | 1 | 10 | 250,186 | 1,191x |
|  |  |  |  |  | 2 | 17.5 | 285,210 | 1,351x |
|  |  |  |  |  | 3 | 23.9 | 525,095 | 2,438x |
| **Case #5**  **PT (50)** | FFPE | 95 | 1.2 | 250 | 1 | 5.8 | 177,697 | 839x |
|  |  |  |  |  | 2 | 23.8 | 249,521 | 1,153x |
|  |  |  |  |  | 3 | 7.8 | 505,555 | 4,896x |
| **Case #5 M(HEP) (29)** | FFPE | 57 | 2.3 | 250 | 1 | 9.8 | 233,353 | 1,088x |
|  |  |  |  |  | 2 | „overload“ | 257,163 | 1,188x |
|  |  |  |  |  | 3 | 7.9 | 560,919 | 2,616x |
| **Case #5 M(PUL) (32)** | FFPE | 37 | 2.6 | 184 | 1 | 5.9 | 173,534 | 812x |
|  |  |  |  |  | 3 | 87.2 | 240,298 | 1,095x |
| **Case #5 M(PUL) (33)** | FFPE | 57 | 2.4 | 250 | 1 | 2.4 | 108,955 | 500x |
|  |  |  |  |  | 3 | 10.2 | 463,667 | 1,997x |
| **Case #6**  **PT (36)** | FFPE | 57 | 2.8 | 250 | 1 | 2.1 | 74,879 | 329x |
|  |  |  |  |  | 3 | 79.8 | 1,050,216 | 4,472x |
| **Case #6 M(PUL) (39)** | FFPE | 45 | 1.8 | 224 | 1 | 2.1 | 86,800 | 396x |
|  |  |  |  |  | 3 | 96.1 | 685,869 | 2,974x |
| **Breast Cancer (5A)** | FFPE | 44 | 3.3 | 220 | 1 | 0.5 | 22,820 | 106x |
|  |  |  |  |  | 2 | 91.6 | 322,349 | 1,356x |
|  |  |  |  |  | 3 | 113.9 | 804,338 | 3,670x |
| **Breast Cancer (5B)** | FFPE | 214 | 3.8 | 250 | 1 | 0 | 249,521 | 65x |
|  |  |  |  |  | 2 | 35.2 | 331,387 | 1,467x |
|  |  |  |  |  | 3 | 90.7 | 293,435 | 1,287x |
| **Breast Cancer (7A)** | FFPE | 234 | 2.1 | 250 | 1 | 0.9 | 47,325 | 224x |
|  |  |  |  |  | 2 | 78.9 | 333,010 | 1,433x |
|  |  |  |  |  | 3 | 26.7 | 756,950 | 3,515x |
| **Breast Cancer (7B)** | FFPE | 269 | 2.7 | 250 | 1 | 1.1 | 54,512 | 261x |
|  |  |  |  |  | 2 | 78.7 | 365,889 | 1,654x |
|  |  |  |  |  | 3 | 109.2 | 712,148 | 3,298x |

**4. Table S2**. **Details of tissue specimens and associated DNA and library processing data.** The table summarizes the sequence library preparation protocols for each DNA sample of the CRC cohort. The tumor cell content of microdissected tissues is given as Tumor %. QC=QC value of qPCR based quality control.

| **Case ID#** | **Tissue specimens** | **Sample ID#** | **Tumor %** | **DNA**  **[ng/µl]** | **QC** | **Input**  **(ng)** | **Library Protocol** | **Mean Coverage** |
| --- | --- | --- | --- | --- | --- | --- | --- | --- |
| 1 | NO | 1 | 0 | 58 | 2.4 | 250 | 2 | 8,071x |
|  | PT | 2 | 60 | 7 | 2.7 | 35 | 4 | 998x |
|  | synM[HEP] | 3 | 30 | 7 | 1.9 | 34 | 5 | 3,137x |
|  | metM[PUL] | 5 | 40 | 34 | 1.3 | 250 | 4 | 3,085x |
| 2 | NO | 6 | 0 | 9 | 2.2 | 47 | 5 | 4,285x |
|  | PT | 7 | 80 | 41 | 1.1 | 207 | 3 | 4,481x |
|  | synM[HEP] | 8 | 70 | 102 | 1.4 | 250 | 3 | 7,344x |
|  | synM[PUL] | 9 | 60 | 81 | 1.2 | 250 | 3 | 4,496x |
|  | synM[PUL] | 11 | 60 | 53 | 0.9 | 250 | 3 | 6,788x |
| 3 | NO | 13 | 0 | 38 | 2.4 | 191 | 3 | 2,037x |
|  | PT | 14 | 80 | 41 | 2.7 | 207 | 3 | 3,136x |
|  | metM[HEP] | 15 | 80 | 90 | 1.9 | 250 | 3 | 3,862x |
|  | metM[PUL] | 16 | 90 | 5 | 1.7 | 24 | 5 | 3,124x |
|  | metM[PUL] | 17 | 90 | 6 | 1.7 | 30 | 5 | 7,063x |
|  | metM[PUL] | 18 | 80 | 6 | 1.4 | 28 | 5 | 3,261x |
| 4 | NO | 21 | 0 | 14 | 1.5 | 71 | 3 | 8,102x |
|  | PT | 22 | 70 | 281 | 0.1 | 250 | 3 | 6,410x |
|  | syn[HEP] | 23 | 70 | 452 | 0.7 | 250 | 3 | 3,008x |
|  | metM[HEP] | 25 | 80 | 25 | 0.1 | 127 | 3 | 4,071x |
|  | metM[PUL] | 49 | 80 | 83 | 2.3 | 250 | 3 | 3,229x |
| 5 | NO | 28 | 0 | 50 | 2.2 | 250 | 3 | 4,282x |
|  | PT | 50 | 70 | 95 | 1.2 | 250 | 3 | 8,608x |
|  | synM[HEP] | 29 | 60 | 57 | 2.3 | 250 | 3 | 4,599x |
|  | synM[PUL] | 32 | 70 | 37 | 2.6 | 183 | 3 | 1,927x |
|  | synM[PUL] | 33 | 80 | 57 | 2.4 | 250 | 3 | 3,512x |
| 6 | NO | 34 | 0 | 128 | 3.0 | 250 | 3 | 4,155x |
|  | PT | 36 | 80 | 57 | 2.8 | 250 | 3 | 7,883x |
|  | metM[HEP] | 38 | 10 | 48 | 2.1 | 240 | 3 | 6,425x |
|  | metM[PUL] | 39 | 90 | 45 | 1.8 | 224 | 3 | 5,230x |
| 7 | NO | 40 | 0 | 77 | 1.8 | 250 | 3 | 3,450x |
|  | PT | 41 | 60 | 92 | 1.1 | 250 | 3 | 6,720x |
|  | synM[HEP] | 42 | 70 | 89 | 1.9 | 250 | 3 | 8,949x |
|  | metM[HEP] | 44 | 70 | 24 | 2.1 | 120 | 3 | 9,552x |
|  | metM[HEP] | 45 | 70 | 244 | 3.0 | 250 | 3 | 9,027x |
|  | metM[PUL] | 47 | 90 | 47 | 0.1 | 236 | 3 | 8,676x |
| 8 | NO | 54 | 0 | 173 | 2.2 | 250 | 3 | 5,933x |
|  | PT | 55 | 80 | 45 | 1.1 | 226 | 3 | 6,859x |
|  | synM[HEP] | 56 | 80 | 28 | 1.1 | 142 | 3 | 7,163x |
|  | synM[PUL] | 58 | 90 | 65 | 1.8 | 250 | 3 | 7,856x |
|  | metM[PUL] | 59 | 70 | 21 | 0.9 | 107 | 3 | 7,696x |
| 9 | NO | 61 | 0 | 20 | 1.4 | 100 | 5 | 8,410x |
|  | PT | 62 | 70 | 67 | 1.7 | 250 | 4 | 4,324x |
|  | synM[HEP] | 63 | 80 | 82 | 0.1 | 250 | 4 | 5,968x |
|  | metM[PUL] | 64 | 70 | 15 | 1.0 | 73 | 5 | 4,558x |
|  | metM[PUL] | 66 | 70 | 46 | 1.2 | 229 | 5 | 7,993x |
|  | metM[PUL] | 68 | 80 | 103 | 2.3 | 250 | 3 | 4,430x |
| 10 | NO | 70 | 0 | 39 | 1.3 | 250 | 4 | 3,477x |
|  | PT | 71 | 60 | 10 | 1.9 | 49 | 5 | 5,024x |
|  | metM[HEP] | 73 | 70 | 151 | 3.0 | 250 | 3 | 8,915x |
|  | metM[PUL] | 74 | 70 | 60 | 0.9 | 250 | 3 | 8,314x |
| 11 | NO | 75 | 0 | 85 | 2.7 | 250 | 3 | 6,742x |
|  | PT | 76 | 70 | 45 | 2.2 | 226 | 3 | 9,873x |
|  | synM[HEP] | 77 | 80 | 100 | 0.9 | 250 | 3 | 6,423x |
|  | metM[PUL] | 78 | 80 | 9 | 0.4 | 45 | 5 | 5,518x |
|  | metM[PUL] | 80 | 80 | 37 | 0.1 | 183 | 5 | 8,217x |
| 12 | NO | 87 | 0 | 71 | 1.4 | 250 | 4 | 7,441x |
|  | PT | 88 | 80 | 11 | 1.1 | 250 | 4 | 3,391x |
|  | synM[HEP] | 89 | 80 | 83 | 2.4 | 250 | 3 | 6,962x |
|  | metM[PUL] | 91 | 80 | 32 | 1.4 | 159 | 3 | 8,881x |
|  | metM[PUL] | 92 | 80 | 61 | 2.4 | 250 | 3 | 6,876x |
| 13 | NO | 94 | 0 | 134 | 1.3 | 250 | 4 | 6,987x |
|  | PT | 95 | 80 | 64 | 1.6 | 250 | 4 | 4,882x |
|  | synM[HEP] | 97 | 20 | 9 | 2.0 | 43 | 5 | 3,906x |
|  | synM[PUL] | 98 | 20 | 17 | 1.3 | 34 | 4 | 4,453x |
|  | synM[PUL] | 99 | 20 | 6 | 2.2 | 31 | 5 | 4,240x |
| 14 | NO | 100 | 0 | 14 | 1.6 | 70 | 3 | 5,859x |
|  | PT | 101 | 70 | 55 | 0.8 | 250 | 3 | 6,553x |
|  | synM[HEP] | 102 | 70 | 22 | 0.9 | 250 | 3 | 6,295x |
|  | synM[HEP] | 103 | 70 | 28 | 1.1 | 139 | 3 | 6,540x |
|  | synM[PUL] | 104 | 80 | 6 | 2.9 | 30 | 3 | 1,992x |

**5. Table S3**. **Validation of variants detected by tNGS by dideoxy sequencing.** Validation was for 27 DNA samples of 8 CRC cases including: 5 gene, 13 sequence variants, 2 wildtype sequences (total 40 sequencing results). Of these, 38/40 (95%) were validated and 2/40 (5%) showed poor quality sequences.

| **Case ID#** | **Sample ID#** | **Gene** | **Exon** | **Codon / variant** | **AA change** | **Allele frequency (%)** | **Confirmed** |
| --- | --- | --- | --- | --- | --- | --- | --- |
| **2** | 7 | KRAS | 2 | gGt/gTt | G12V | 53.59 | Y |
|  | 8 | KRAS | 2 | gGt/gTt | G12V | 89.98 | Y |
|  | 9 | KRAS | 2 | gGt/gTt | G12V | 47.32 | Y |
|  | 11 | KRAS | 2 | gGt/gTt | G12V | 32.82 | Y |
|  | 7 | SMAD4 | 9 | Cgc/Tgc | R361C | 24.42 | Y |
|  | 8 | SMAD4 | 9 | Cgc/Tgc | R361C | 83.88 | Y |
|  | 9 | SMAD4 | 9 | Cgc/Tgc | R361C | 36.21 | Y |
|  | 11 | SMAD4 | 9 | Cgc/Tgc | R361C | 26.75 | Y |
| **4** | 22 | TP53 | 10 | Cgc/Tgc | R337C | 22.13 | Y |
|  | 22 | TP53 | 5 | Cgc/Tgc | R156C | 36.05 | Y |
|  | 23 | TP53 | 10 | Cgc/Tgc | R337C | 28.13 | Y |
|  | 23 | TP53 | 5 | Cgc/Tgc | R156C | 33.02 | Y |
|  | 25 | TP53 | 10 | Cgc/Tgc | R337C | 20.75 | Y |
|  | 25 | TP53 | 5 | Cgc/Tgc | R156C | 39.74 | Y |
|  | 49 | TP53 | 10 | Cgc/Tgc | R337C | 22.52 | N |
|  | 49 | TP53 | 5 | Cgc/Tgc | R156C | 37.67 | Y |
| **5** | 29 | PIK3CA | 9 | Gaa/Aaa | E542K | 23.42 | Y |
|  | 32 | PIK3CA | 9 | Gaa/Aaa | E542K | 13.78 | N |
|  | 33 | PIK3CA | 9 | Gaa/Aaa | E542K | 17.24 | Y |
|  | 50 | PIK3CA | 9 | Gaa/Aaa | E542K | 25.94 | Y |
| **6** | 36 | KRAS | 2 | gGt/gTt | G12V | 22.85 | Y |
|  | 38 | KRAS | 2 | cag | WT | 0 | Y |
|  | 39 | KRAS | 2 | gGt/gTt | G12V | 26.73 | Y |
|  | 36 | SMAD4 | 6 | Cag/Tag | Q250* | 27.09 | Y |
|  | 38 | SMAD4 | 6 | Cag | WT | 0 | Y |
|  | 39 | SMAD4 | 6 | Cag/Tag | Q250* | 45.69 | Y |
| **9** | 62 | TP53 | 8 | Cgg/Tgg | R282W | 64.03 | Y |
|  | 63 | TP53 | 8 | Cgg/Tgg | R282W | 81.53 | Y |
|  | 64 | TP53 | 8 | Cgg/Tgg | R282W | 69.63 | Y |
| **10** | 73 | TP53 | 5 | gTc/gGc | V157G | 49.73 | Y |
| **11** | 76 | TP53 | 8 | Cgt/Tgt | R273C | 14.48 | Y |
| **12** | 88 | KRAS | 2 | gGt/gCt | G12A | 99.74 | Y |
|  | 92 | KRAS | 2 | gGt/gCt | G12A | 19.63 | Y |
| **13** | 96 | KRAS | 2 | gGt/gAt | G12D | 19.80 | Y |
|  | 97 | KRAS | 2 | gGt/gTt | G12V | 16.44 | Y |
|  | 98 | KRAS | 2 | gGt/gTt | G/V | 5.62 | Y |
|  | 99 | KRAS | 2 | gGt/gTt | G12V | 25.72 | Y |
| **14** | 101 | NRAS | 3 | cAa/cGa | Q61R | 44.31 | Y |
|  | 103 | NRAS | 3 | cAa/cGa | Q61R | 44.74 | Y |
|  | 101 | TP53 | 5 | cGc/cAc | R175H | 52.35 | Y |
|  | 102 | TP53 | 5 | cGc/cAc | R175H | 69.24 | Y |
|  | 103 | TP53 | 5 | cGc/cAc | R175H | 75.8 | Y |

**6. Figure S1**. **Quantity and quality of tNGS libraries of the standard and adapted library protocols. A)** Comparison of the standard tNGS library protocol of DNA samples from three carcinomas (non-small cell lung carcinoma, colorectal carcinoma and breast carcinoma) with matched Fresh-frozen and Formalin-fixed and Paraffin-embedded tissue specimens (FFPE). **B)** Comparison of standard and adapted tNGS library protocols of DNA samples from two representative carcinomas (breast, colorectal carcinomas) upon same DNA input. **A), B)** Graphs represent electropherograms and quantification (library concentration in nM) of the tNGS libraries (Bioanalyzer 2100, Agilent Technologies, Santa Clara, USA). Input DNA quantities as well as DNA quality given as “QC” as measured by the “FFPE QC KIT” (Illumina, San Diego, USA) are given. Sequencing data are provided as cluster density and mean coverage. Refer to main text and Supplementary Table S1 for further details.

**
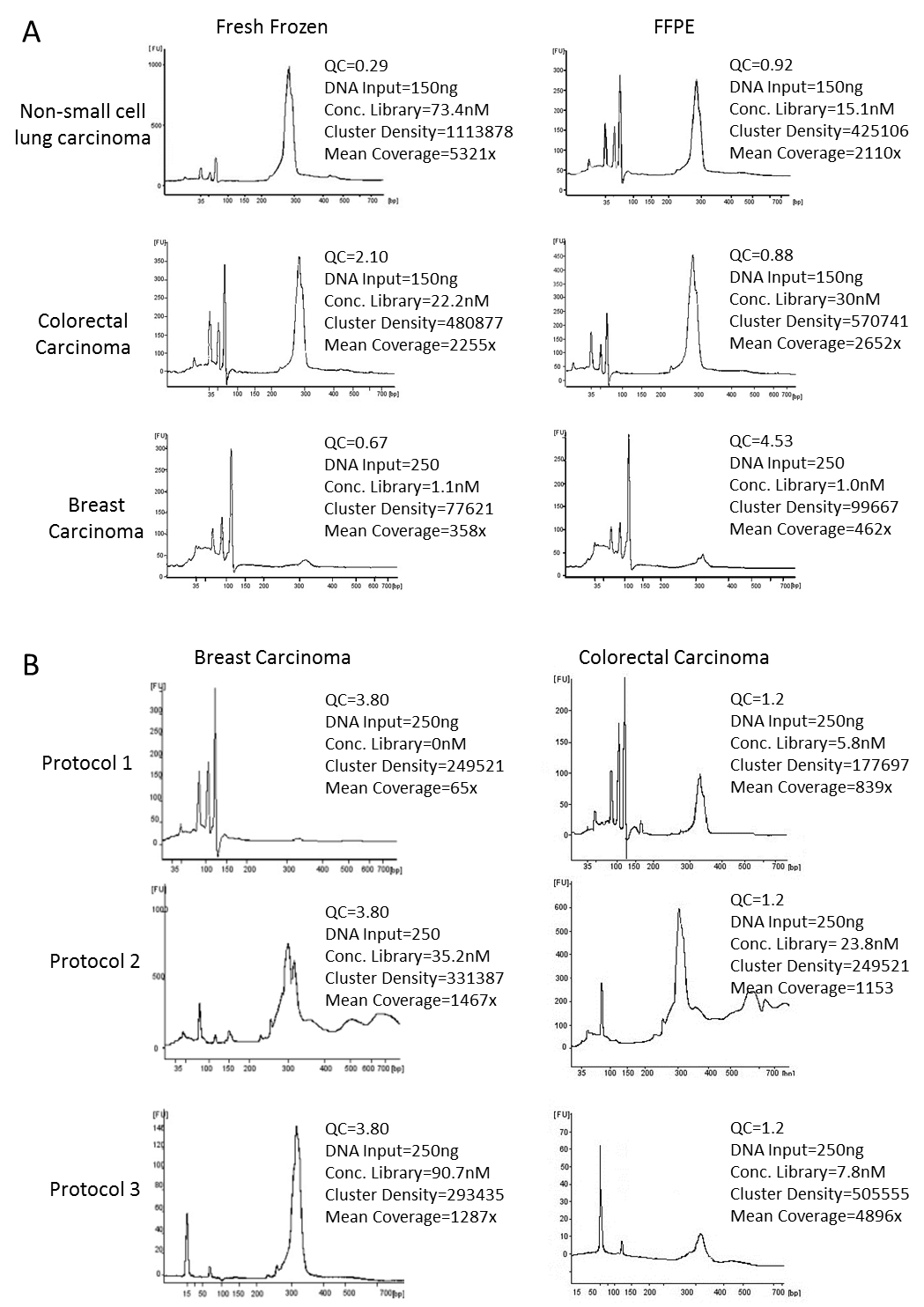
**

**7. Figure S2**. ***De novo* FBXW7 sequence variants detected in colorectal carcinoma liver and/or lung metastases.** **A)** Documentation of each FBXW7 mutation detected in this study allocated to the COSMIC “hotspot” panel**. B)** List of sequence variants detected in CRC cases and tissue specimens of this study. Refer to main text for details.


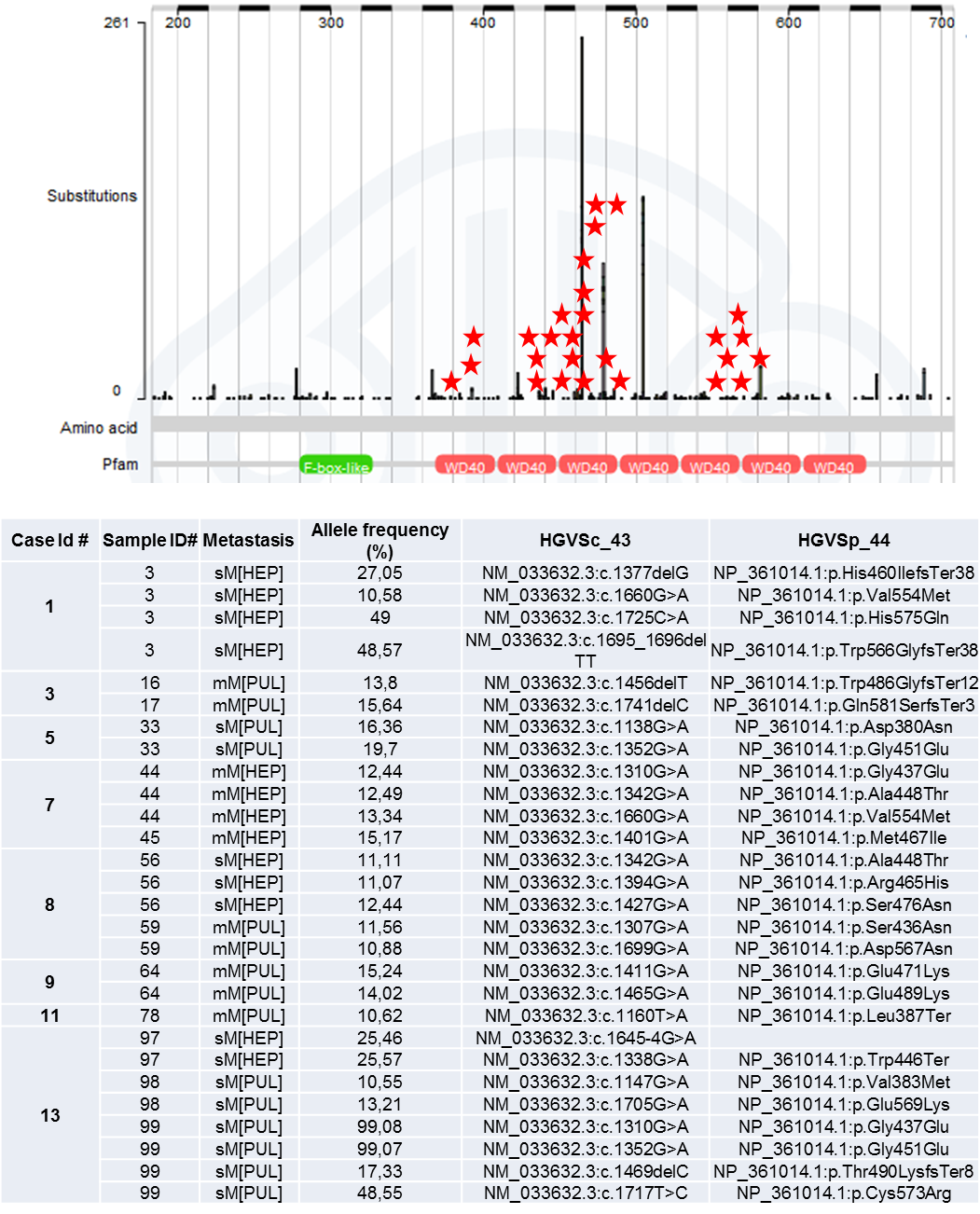

Supplement: Additional file 1: — Text S1. Establishment of library preparation from DNA samples derived from different origin tissue specimens. Adaptations of the tNGS library protocol. Table S1. Modifications to tNGS library protocol of low input or poorer quality DNA improves sequencing performance. Table S2. Details of tissue specimens and associated DNA and library processing data. Table S3. Validation of variants detected by tNGS by dideoxy sequencing. Figure S1. Quantity and quality of tNGS libraries of the standard and adapted library protocols. Figure S2. De novo FBXW7 sequence variants detected in colorectal carcinoma liver and/or lung metastases. (DOCX 556 kb) [file 12943_2016_549_MOESM1_ESM.docx]
